# Evaluation of a Positive Youth Development Program for Adolescents with Greater Psychosocial Needs: Integrated Views of Program Implementers

**DOI:** 10.1100/tsw.2010.160

**Published:** 2010-10-01

**Authors:** Daniel T. L. Shek, Cecilia M. S. Ma, Rachel C. F. Sun

**Affiliations:** ^1^ Department of Applied Social Sciences, Hong Kong Polytechnic University, Hong Kong, China; ^2^ Public Policy Research Institute, The Hong Kong Polytechnic University, Hong Kong, China; ^3^ Department of Sociology, East China Normal University, Shanghai, China; ^4^ Division of Adolescent Medicine, Department of Pediatrics, Kentucky Children's Hospital, University of Kentucky College of Medicine, Lexington, Kentucky, USA; ^5^ Faculty of Education, University of Hong Kong, Hong Kong, China

**Keywords:** subjective outcome evaluation, secondary data analysis, program evaluation, Project P.A.T.H.S., Chinese adolescents with greater psychosocial needs

## Abstract

To help adolescents with greater psychosocial needs, the Tier 2 Program of the Project P.A.T.H.S. (Positive Adolescent Training through Holistic Social Programmes) was designed and implemented by school social workers and teachers. Based on subjective outcome evaluation data collected from the program participants (n = 2,542) in 49 schools, program implementers were invited to write down five conclusions based on an integration of the evaluation findings. With reference to 245 conclusions included in the 49 evaluation reports, secondary data analyses showed that most of the conclusions concerning perceptions of the Tier 2 Program, instructors, and program effectiveness were positive. In addition, difficulties encountered and recommendations for program improvement were highlighted. In conjunction with previous evaluation findings, the present study suggests that the Tier 2 Program was well received and was perceived to be beneficial to the development of adolescents with greater psychosocial needs.

### INTRODUCTION

Secondary analysis refers to the use of an existing dataset that was not fully analyzed in the primary study in order to answer new research questions[1,2]. Secondary analysis of qualitative data makes use of data collected by other researchers[3] or conducts in-depth analyses of an existing dataset. Studies that employ this approach can be found in various fields, including the food environment[4], environmental management[5], and health care[3]. However, a survey of the literature shows that not much work has been done in the fields of social work and education with school students. In fact, researchers emphasize the use of secondary analysis research to expand the body of knowledge in the field of developmental psychology[6,7]. For example, Friedman[8] suggested that developmental psychologists should "consider using existing data sets as one method to answer their scientific questions…the use of such data sets is an important part of behavioral sciences research, as it affords the investigation of an even broader set of research questions than those that originally motivated the study" (p. 384).

Traditionally, program evaluators should be "detached" from the program implementation when conducting evaluative research[9]. Also, frontline service providers are expected to keep a distance from the evaluators, who are perceived as experts judging on their performance and ways of thinking and behavior[10]. Moreover, program implementers would not be asked to conduct program evaluation because of the concern that collection of data for evaluation would distract their effort to implement the program and service efficiently[11]. Nevertheless, there is a growing emphasis on the importance of engaging different stakeholders, especially program implementers, in the evaluation process.

There are several reasons for engaging program implementers in the evaluation process. These include facilitation of the full utilization of evaluation findings, integration of divergent values and views of different stakeholders during the decision-making process, and empowerment of those marginalized or silent groups not involved previously in the evaluation process[12,13,14]. By engaging the program implementers in the evaluation process, the likelihood of capturing a better picture of the intended outcomes of the program would increase. For example, the evaluation data would help to identify challenges related to the implementation of the program and evaluation strategies, so that evaluators and program implementers could revisit the measures used to assess the targeted outcomes of the program and discuss the need for additional measures or process data.

In different evaluation paradigms, researchers have commonly upheld the principle that the views of different parties should be assessed in order to reveal the complexity of the phenomena under evaluation. In another words, it can be argued that it would be incomplete if the program implementers' views were left out. For example, in the utilization-focused evaluation paradigm, it is argued that as different stakeholders are involved in the evaluation process, program implementers' views are legitimately covered. Similarly, based on the standards of the Joint Committee on Standards for Educational Evaluation[15], identification of the stakeholders (Utility Standard 1) involving complete and fair assessment (Proprietary Standard 5) would be important. According to these standards, program implementers' views and assessments should be taken into account. Unfortunately, while there are arguments supporting the involvement of program implementers in the evaluation process, a survey of the evaluation literature shows that program implementers are rarely invited to give their views under the subjective outcome evaluation approach (i.e., client satisfaction approach). Although views of the participants are the ultimate concern[16], it is not a common practice to examine the views of the program implementers[17].

The Project P.A.T.H.S. (**P**ositive **A**dolescent **T**raining through **H**olistic **S**ocial Programmes) is a large-scale positive youth development program designed for junior secondary school students (Secondary 1 to 3, i.e., Grades 7 to 9) in Hong Kong. There are two tiers of program in this project. The Tier 1 Program targets all students who join the program in a particular form (i.e., universal prevention initiative). Through the use of a structured curriculum, students learn psychosocial competencies based on 15 positive youth development constructs[18]. On the other hand, the Tier 2 Program is designed especially for students with greater psychosocial needs in different psychosocial domains (i.e., selective prevention initiative). After completion of the Tier 2 Program, program participants were invited to complete a subjective outcome evaluation form (Form C) and written informed consent was collected from them. Based on the subjective outcome evaluation data collected, the responsible program implementers in each school were required to complete an evaluation report, where they were asked to write down five conclusions regarding the program and its effectiveness.

The primary research goal of the present study was to assess the effectiveness of the Tier 2 Program of the Project P.A.T.H.S. based on the conclusions drawn by the program implementers of programs designed for Secondary 2 students with greater psychosocial needs via secondary data analyses. The aim was to explore whether the findings as reported in previous studies[19] could be replicated.

## METHODS

### Dataset for Secondary Data Analyses

Forty-nine schools joined the Tier 2 Program of the Project P.A.T.H.S. in the Experimental Implementation Phase in the 2006/07 school year. Among these schools, 2,542 participants were involved, of which 2,439 Secondary 2 students were identified by teachers, parents, and/or self-administered questionnaires as having greater psychosocial needs, and were invited to join the Tier 2 Program. The remaining 103 participants were the parents and teachers of those students identified as having greater psychosocial needs and they were also invited to participate in the Tier 2 Program. The mean number of participants joining the Tier 2 Program per school was 51.88 (range: 17–240). The average number of sessions provided per school (normally 1.5–3 h per session) was 22.63 (range: 1–62). After completion of the Tier 2 Program, a total of 1,898 participants (mean = 38.73 participants per school, range: 8–199) were invited to respond to the Subjective Outcome Evaluation Form (Form C) developed by Shek and colleagues[20]. The overall response rate was 74.67%. There were three factors contributing to this response rate: (1) some participants withdrew from the program early, (2) some participants were absent from the last session and did not complete the evaluation form, and (3) some schools did not invite the adult participants to respond to the evaluation form.

The Subjective Outcome Evaluation Form (Form C) was used to measure the perceptions of the Tier 2 Program. There are seven parts in this evaluation form:

1. Participants' perceptions of the program, such as program arrangement, quality of service, appropriateness of the program, and interaction among the participants (eight items)
2. Participants' perceptions of the workers, such as the preparation of the workers, professional attitude and knowledge, and interaction with the participants (eight items)
3. Participants' perceptions of the effectiveness of the program, such as promotion of problem-solving skills, behavioral modification, and positive change (eight items)
4. Things that the participants appreciated most (open-ended question)
5. Opinion about the workers (open-ended question)
6. Things that the participants learned from the program (open-ended question)
7. Areas that require improvement (open-ended question)

To facilitate the program evaluation, the research team developed an evaluation manual with standardized instructions for collecting the subjective outcome evaluation data. In addition, adequate training was provided to the social workers during 20-h workshops on how to collect and analyze the data using Form C.

Based on the evaluation data collected in each school, the responsible worker was required to complete an evaluation report, where the quantitative and qualitative findings based on Form C were summarized and described. In the last section of the report, the worker preparing the report in each school was requested to write down five conclusions regarding the program and its effectiveness based on all the available information and experiences, which can give an overall picture regarding the perceived effectiveness of the Tier 2 Program. The evaluation reports were then submitted to The Hong Kong Jockey Club Charities Trust for the purpose of program monitoring. Based on the submitted evaluation reports (i.e., primary data), further aggregation and syntheses of the findings were carried out in this study.

### Data Analyses

The data were analyzed using general qualitative analyses techniques[21] by two trained research assistants, of which one has a Bachelor's Degree in Psychology and another has a Bachelor's Degree in Social Work. The final coding and categorization was further cross-checked by a research colleague with a Master's Degree in Social Work. There were two steps in the data analysis process. First, raw codes were developed for words, phrases, and/or sentences that formed meaningful units in each conclusion at the raw responses level. Second, the codes were further combined to reflect higher-order attributes at the category of codes level. For example, the response of "like the program" at the raw response level could be subsumed under the category of "satisfaction level", which could be further subsumed under the broad theme of "stakeholders' view on the program" (see Table 1).

In the present qualitative data analyses, since the first author designed the P.A.T.H.S. program, he was conscious of his own biases and expectations of the program's effectiveness. As such, the first author was not directly involved in the data analyses. In addition, in order to minimize the possible biases involved, both intra- and inter-rater reliability on the coding were calculated. For intrarater reliability, the two research assistants primarily responsible for coding coded 20 randomly selected responses without looking at the original codes given after checking by the first author. For inter-rater reliability, another two research assistants who had not been involved in the data analyses, both with a Master's Degree, coded the same 20 randomly selected responses without knowing the original codes given at the end of the scoring process.

Following the principles of qualitative analyses proposed by Shek and colleagues[22], the following attributes of the study regarding data collection and analyses were highlighted. First, a general qualitative orientation was adopted. Second, the sources of data (e.g., number of participants) for analyses were described. Third, the issues of biases and ideological preoccupation were addressed. Fourth, inter- and intrarater reliability information was presented. Fifth, the categorized data were kept by a systematic filing system in order to ensure that the findings were auditable. Finally, possible explanations, including alternative explanations, were considered.

### RESULTS

Based on the 245 conclusions in 49 reports, a total of 441 meaningful units were extracted. These raw responses were further grouped into several categories, including views of the stakeholders on the program (Table 1), views towards instructors (Table 2), perceived effectiveness of the program (Table 3), difficulties encountered (Table 4), and recommendations for improvement (Table 5).

Regarding the conclusions related to the stakeholders' perceptions of the program, results in Table 1 show that most of the responses were positive in nature in the areas of satisfaction, overall impression, program content and format, learning process, program arrangement and implementation, other appreciation, and additional merits. Among the 103 responses, 96 were classified as positive (93.2%). The intra- and inter-rater agreement percentages of the positivity of the coding were 100 and 92.5%, respectively. For the perceptions of the program implementers, findings in Table 2 show that most of the responses were positive in nature. Among the 67 responses, 66 were positive (98.51%). Both the intra- and inter-rater agreement percentages of the positivity of the coding were 100%.

The responses related to perceived effectiveness of the program are shown in Table 3. There were a total of 217 meaningful units and they were categorized in several levels, including societal level, interpersonal level (general interpersonal competence and specific interpersonal competence), personal level (positive self-image, emotional competence, cognitive competence, responsibility, ways to face adversity, goal setting, reflection, behavioral competence, and learning and knowledge) and others. All 217 responses were positive (100%). Both the intra- and inter-rater agreement percentages of the positivity of the coding were 95%.

Table 4 shows the responses related to the difficulties encountered in the implementation of the program. There were 10 responses in this dimension, of which four were related to program and six were related to students' factors. As it was meaningless to categorize the positivity nature of the encountered difficulties, no categorization of the positivity of the responses was conducted. The intra- and inter-rater agreement percentages of the categorization of the coding were 80 and 90%, respectively.

**TABLE 1**
**Responses Related to the Stakeholders' Views on the Program**

| Category | Descriptions | Positive | Neutral | Negative | Undecided | Total | Subtotal |
|---|---|---|---|---|---|---|---|
| Satisfaction Level | Liked the program very much | 4 | | | | 4 | 21 |
| | Liked the program | 14 | | | | 14 | |
| | Fair | | 3 | | | 3 | |
| Overall Impression | Meaningful | 1 | | | | 1 | 10 |
| | Could achieve the objectives | 2 | | | | 2 | |
| | Could meet students' needs | 6 | | | | 6 | |
| | Could provide more comprehensive services for students | 1 | | | | 1 | |
| Program Content and Format | Positive views on program content | 5 | | | | 5 | 30 |
| | Positive views on program format | 2 | | | | 2 | |
| | Diversified activities | 3 | | | | 3 | |
| | Students appreciated particular activities | 17 | | | | 17 | |
| | Provided opportunities to serve the community | 2 | | | | 2 | |
| | Emphasized thinking and discussion | 1 | | | | 1 | |
| Learning Process | Happy | 1 | | | | 1 | 6 |
| | Students' active participation | 5 | | | | 5 | |
| Program Arrangement and Implementation | Appreciated program arrangement and quality | 11 | | | | 11 | 18 |
| | Good relationship between students and workers facilitated the implementation | 3 | | | | 3 | |
| | Whole school cooperation facilitated the implementation | 4 | | | | 4 | |
| Other Appreciation | Would recommend the program to others | 3 | | | | 3 | 6 |
| | Would join the program again | 2 | | | | 2 | |
| | The program was worth to continue | 1 | | | | 1 | |
| Additional Merits | Offered a platform for social workers to provide tailor-made services for students | 2 | | | | 2 | 8 |
| | Offered a platform for social workers to understand students' growth | 2 | | | | 2 | |
| | Facilitated the case work of school social workers | 1 | | | | 1 | |
| | Connection between teachers and school social workers allowed teachers to further reassure students | 1 | | | | 1 | |
| | Could stimulate students' interests and motivation on learning | 2 | | | | 2 | |
| Others | Others | | 2 | 1 | 1 | 4 | 4 |
| **Total Responses** | | **96** | **5** | **1** | **1** | **103** | **103** |

*Note*: Twenty coded raw descriptors were randomly selected for examining intra- and inter-rater reliability. The raters were required to code the randomly selected descriptors into four categories (i.e., positive, neutral, negative, and undecided) without knowing the original codes given.

**TABLE 2**
**Responses on Views towards Instructors**

| Category | Descriptions | Positive | Neutral | Negative | Undecided | Total | Subtotal |
|---|---|---|---|---|---|---|---|
| General Appreciation | Appreciated attitude | 11 | | | | 11 | 33 |
| | Appreciated performance | 16 | | | | 16 | |
| | Appreciated implementation skills | 6 | | | | 6 | |
| Specific Appreciation | Devoted | 1 | | | | 1 | 32 |
| | Well prepared for the program | 5 | | | | 5 | |
| | Cared about students | 8 | | | | 8 | |
| | Had professional knowledge | 4 | | | | 4 | |
| | Had interactions with students | 3 | | | | 3 | |
| | Maintained good relationship with students | 1 | | | | 1 | |
| | Instructors' attitude and performance enhanced students' learning | 10 | | | | 10 | |
| Others | Gained students’ respect | 1 | | | | 1 | 2 |
| | Failed to take care of all students | | | 1 | | 1 | |
| **Total Responses** | | **66** | **0** | **1** | **0** | **67** | **67** |

*Note*: Twenty coded raw descriptors were randomly selected for examining intra- and inter-rater reliability. The raters were required to code the randomly selected descriptors into four categories (i.e., positive, neutral, negative, and undecided) without knowing the original codes given.

Responses on recommendations to the program are presented in Table 5. There were 44 meaningful units classified into program content and program implementation. As it was meaningless to categorize the positivity nature of the recommendations, no categorization of the positivity of the responses was conducted. The intra- and inter-rater agreement percentages of the categorization of the coding were 92.5 and 70%, respectively.

### DISCUSSION

Consistent with the findings reported by Siu and Shek[19], the present study showed that the participants perceived the qualities of program content, instructors, and effectiveness of the program positively. In conjunction with the evaluation findings based on Form C, the available evidence suggests that different stakeholders, including program participants and implementers, perceived the Tier 2 Program in a favorable light and saw the program as beneficial to the students. In view of the rising adolescent developmental problems in Hong Kong, the present study suggests that the Tier 2 Program of the Project P.A.T.H.S. is beneficial to the adolescents with greater psychosocial needs. From a public health perspective, to prevent one’s psychosocial well-being from deteriorating, early identification of and treatment for adolescents with greater psychosocial needs is of paramount importance. The present findings are important because there is growing research evidence showing that risk behavior in adolescents in Hong Kong is intensifying.

The present findings are consistent with those based on the Tier 1 Program in which the participants generally had positive perceptions of the quality of program content, instructors, and effectiveness of the program. These findings generally suggest that both Tier 1 and Tier 2 programs of the Project P.A.T.H.S. are generally well received by the program participants. The positive findings underscore the utility of a curricular-based approach in promoting positive adolescent development in Hong Kong. This observation is significant as there are few validated positive youth development programs in Hong Kong.

**TABLE 3**
**Responses on Perceived Effectiveness**

| Category/Subcategory | | Descriptions | Positive | Neutral | Negative | Undecided | Total | Subtotal |
|---|---|---|---|---|---|---|---|---|
| Societal Level | | Enhanced social responsibility and participation | 11 | | | | 11 | 11 |
| Interpersonal Level | General Interpersonal Competence | Improved interpersonal relationship | 20 | | | | 20 | 56 |
| | | Enhanced instructors and students relationship | 3 | | | | 3 | |
| | | Enhanced peer relationship | 7 | | | | 7 | |
| | Specific Interpersonal Competence | Promoted cooperation with others | 18 | | | | 18 | |
| | | Appreciated others | 2 | | | | 2 | |
| | | Promoted mutual care/altruism | 6 | | | | 6 | |
| Personal Level | Positive Self-Image | Enhanced students' development | 8 | | | | 8 | 140 |
| | | Had impact on students' development | 27 | | | | 27 | |
| | | Enhanced self-presentation skills | 3 | | | | 3 | |
| | | Promoted self-efficacy | 5 | | | | 5 | |
| | | Promoted self-esteem | 7 | | | | 7 | |
| | | Enhanced self-confidence | 12 | | | | 12 | |
| | | Established positive self-identity | 10 | | | | 10 | |
| | | Enhanced self-understanding | 5 | | | | 5 | |
| | | Self-enhancement | 13 | | | | 13 | |
| | | Became active | 7 | | | | 7 | |
| | | Explored potentials/breakthrough | 6 | | | | 6 | |
| | | Became independent | 2 | | | | 2 | |
| | Emotional Competence | Promoted emotional control | 2 | | | | 2 | |
| | Cognitive Competence | Promoted analytical ability | 1 | | | | 1 | |
| | Responsibility | Enhanced sense of responsibility | 2 | | | | 2 | |
| | Ways to Face Adversity | Cultivation of resilience | 6 | | | | 6 | |
| | Goal Setting | Promoted goal setting | 4 | | | | 4 | |
| | Reflection | Facilitated life reflection | 4 | | | | 4 | |
| | Behavioral Competence | Enhanced skills of problem solving and conflict management | 7 | | | | 7 | |
| | | Behavioral improvements | 2 | | | | 2 | |
| | Learning and Knowledge | Widened horizons | 3 | | | | 3 | |
| | | Enhanced knowledge and application of multiple intelligence | 4 | | | | 4 | |
| Others | | Other positive impacts | 6 | | | | 6 | 10 |
| | | Enhanced belongingness and contribution to school | 4 | | | | 4 | |
| **Total Responses** | | | **217** | **0** | **0** | **0** | **217** | **217** |

*Note*: Twenty coded raw descriptors were randomly selected for examining intra- and inter-rater reliability. The raters were required to code the randomly selected descriptors into three categories (i.e., societal, interpersonal, and personal) without knowing the original codes given.

**TABLE 4**
**Responses on Encountered Difficulties**

| Category | Descriptions | Total | Subtotal |
|---|---|---|---|
| Program | Clashed with other school activities | 2 | 4 |
| | Time constraint | 2 | |
| Students' Factors | Students lacked commitment | 3 | 6 |
| | Students were passive | 1 | |
| | Weak cohesion among students | 2 | |
| **Total Responses** | | **10** | **10** |

*Note*: Ten coded raw descriptors were randomly selected for examining intra- and inter-rater reliability. The raters were required to code the randomly selected descriptors into two categories (i.e., program and students' factor) without knowing the original codes given.

A major strength of the present study is the involvement of the program implementers in the evaluation process. As program implementers (i.e., teachers and school social workers) worked in the school and interacted closely with the students, they were able to detect sensitive issues and maintain constant communication with the students easily. They are considered as "valid local data" (p. 92)[23], as they possess specific knowledge that can be valuable in the evaluation process. Their unique observations and insights would be essential for program modification and implementation as it makes the quality of evaluation more holistic and comprehensive[24]. Patton[25] argued that the creation of "personal factor" through the involvement of program implementers in the evaluation process would likely to increase the utilization of the evaluation data. Eventually, they would appreciate the things that can be learned from evaluation and the impact of this continuous process on their skills of systematic inquiry[26,27]. Obviously, inviting program implementers to write down five conclusions reflects the spirit of involving various stakeholders and respecting their engagement experiences.

Researchers highlight the importance of constant and open dialogue among stakeholders and program implementers[9]. This not only provides ongoing updates on the project, it also allows implementers to make substantial adjustments when needed. As pointed out by O'Brecht[28], "the best designed organizational structure may fail to produce a high-quality, useful evaluation product if the persons involved do not understand the evaluation process or are not able to function effectively as team players" (p. 146). The participation of the stakeholders would also promote empowerment and increase their capacity in the evaluation process[14].

Unlike the traditional evaluative methods that adopt positivistic ideals, the role of the evaluators in participatory research is no longer to be "detached" from the implementation process[9]. Under the framework of utilization-focused evaluation, evaluators worked with program staff as well as the stakeholders[29]. This leads to "true" participatory behavior by engaging different parties in the program evaluation process. This not only contributes to the establishment of trust, rapport, and credibility between evaluators and stakeholders, but also allows the cultivation of equitable relationships and power in the program implementation and evaluation processes[30,31]. The changes that occurred after the evaluation process would reinforce the stakeholders' commitment to employ the data for program improvement as well as their future dedication to evaluation[32].

Another strength of this study is the use of secondary analysis of qualitative data based on the stakeholders' perspective in the fields of social work and education. Using the existing dataset would "maximize the utilization of data and recognize the value of participants' contributions to the research" (p. 101)[3], thereby increasing their engagement and sense of ownership towards the program[24]. Therefore, the credibility of the entire evaluation process and participant motivation would be established and sustained, as well as "the rigorousness of the evaluation findings" would be preserved. Friedman[8] highlighted the value of secondary analysis as it provides a way to obtain new research answers based on

**TABLE 5**
**Responses on Recommendations to the Program**

| Category | Descriptions | Total | Subtotal |
|---|---|---|---|
| Program Content | More (diverse) activities | 6 | 17 |
| | More whole-form activities to benefit all students | 1 | |
| | Communication skills training should be provided before community services participation | 1 | |
| | Increase the proportion of training camps | 1 | |
| | Provide a room for students to discuss dating and sexual relationships | 1 | |
| | Emphasize dating and sexual education in junior forms | 1 | |
| | Add group sessions | 1 | |
| | Activities should meet students' needs and progress | 2 | |
| | Further considerations on the daily application of skills learned in program | 2 | |
| | Add consolidation sessions for students' sharing and self-reflection | 1 | |
| Program Implementation | Better time arrangement | 4 | 27 |
| | Need good tools | 1 | |
| | Adequate preparation | 1 | |
| | Better program design and arrangement to eliminate labeling effect | 3 | |
| | Teachers' and workers' encouragement and appreciation | 2 | |
| | Good relationship between students and workers | 1 | |
| | Cooperation between school and agency | 1 | |
| | Harmonious atmosphere | 2 | |
| | Aware of group combination | 1 | |
| | Strengthen bonding within systems | 2 | |
| | Develop a reward-punishment scheme | 1 | |
| | Invite students to implement the activities | 1 | |
| | Let students understand the program requirements and expectations clearly | 1 | |
| | Students have self-determination on grouping | 1 | |
| | Foster students' commitment | 1 | |
| | Aware of and better utilize the comments and demands from students | 1 | |
| | Better program arrangement | 3 | |
| **Total responses** | | **44** | **44** |

*Note*: Twenty coded raw descriptors were randomly selected for examining intra- and inter-rater reliability. The raters were required to code the randomly selected descriptors into four categories (i.e., positive, neutral, negative, and undecided) without knowing the original codes given.

data already collected in the original study at a relatively low cost. This is desirable especially to those researchers who have limited resources to conduct original research. Hence, secondary analysis is a valuable, but often neglected, research tool[6].

There are several limitations of the study. First, because of the nature of secondary analysis, it is not possible to have more interactive collaboration with the program implementers. Second, the related findings cannot give us an in-depth understanding of the implementation process due to the brief summary as written by the workers. Third, from the methodology perspective, the validity of this study lies in the assumption that program implementers can make reasonable and fair judgments about the program based on the subjective outcome evaluation findings. This assumption might be met as teachers and social workers in Hong Kong are well trained to conduct evaluative research and practice. Finally, if resources permit, it would be desirable to have further dialogues and discussions with the program implementers who prepare the reports to expand on the meanings of the findings.

Despite these limitations, the present findings are in line with previous findings[33,34,35] that showed that the Project P.A.T.H.S. is well received by the stakeholders and considered as helpful to adolescent development. In particular, the present findings replicate those of a previous study[19] and suggest that there are positive perceptions associated with the Tier 2 Program of the Project P.A.T.H.S. and the program is beneficial to adolescents with greater psychosocial needs in Hong Kong.

Regarding future research direction, there are two issues that should be addressed. First, it would be interesting to ask how program implementation factors contribute to program effectiveness. In the previous studies, it was shown that although program instructors contributed to program success, qualities of program implementers, including passion, commitment, and involvement, were also very important[36,37]. Second, it would be important to examine how subjective outcomes assessed by Form C were related to objective outcome indicators in the long run. Such findings would give some insight to the generalizability of the intervention effect in real-life settings.

### ACKNOWLEDGMENTS

The preparation for this paper and the Project P.A.T.H.S. were financially supported by The Hong Kong Jockey Club Charities Trust.
